# Cross-national aspects of cyberbullying victimization among 14–17-year-old adolescents across seven European countries

**DOI:** 10.1186/s12889-018-5682-4

**Published:** 2018-07-10

**Authors:** Kalliope Athanasiou, Eirini Melegkovits, Elisabeth K. Andrie, Charalampos Magoulas, Chara K. Tzavara, Clive Richardson, Donald Greydanus, Maria Tsolia, Artemis K. Tsitsika

**Affiliations:** 1 Adolescent Health Unit, Second Department of Pediatrics, P. and A. Kyriakou Children’s Hospital, University of Athens, Leoforos Mesogeion 24, 11527 Athens, Greece; 2 Department of Economic and Regional Development, Panteion University of Social and Political Sciences, Athens, Greece; 3 Department of Pediatrics School of Medicine, Western Michigan University, Kalamazoo, MI USA

**Keywords:** Cyberbullying, Cybervictims, Adolescents, Cross-cultural differences

## Abstract

**Background:**

The increasing use of the Internet and social network sites (SNS) has created a new domain of socio-emotional development for adolescents. The aim of this cross-sectional study was to explore cybervictimization across seven European countries, in relation to socio-demographic, Internet use and psychosocial variables.

**Methods:**

A cross-sectional school-based study was conducted in the participating countries: Germany, Greece, Iceland the Netherlands, Poland, Romania and Spain. Anonymous self-completed questionnaires included sociodemographic data, internet usage characteristics, school achievement, parental control, the Internet Addiction Test and Achenbach’s Youth Self-Report.

**Results:**

The highest rate of cyber victimization was found in Romania (37.3%) and the lowest in Spain (13.3%). Multiple logistic regression analyses gave differing results between countries. In Romania, Poland and Germany cyberbullying victimization was associated with SNS use, whereas Internet use was associated with increased odds of cybervictimization only in Romania. Cybervictimization was associated with greater internalizing behavior problems in all countries analysed, and with externalizing problems in all except Romania.

**Conclusions:**

Cyberbullying victimization is an on-going problem, which is subject to country-specific socio-demographic factors and diverse patterns of current Internet use and its development. Preventive measures should emphasize the integration of Internet communication technology education in educational contexts, and focus on the consistent association between cybervictimization and internalizing and externalizing difficulties.

## Background

The increased use of the Internet and contemporary technological devices as primary means of adolescent socialization has become a major issue of concern. Although Internet technology promotes socialization with peers through increasing opportunities for communication, its use has been linked to an array of online risks, including cyberbullying victimization [1, 2]. Specifically, ownership of a social network site (SNS) profile has been associated with higher incidences of cyberbullying [3] and cybervictimization [4, 5], especially in relation to the surge in the use of mobile phones for continual SNS access [6, 7].

### Definition and prevalence of Cybervictimization

“Cyberbullying” is used here to describe intentional aggressive behaviors with the purpose of hurting the victim, distinguished from traditional bullying in that it occurs through technological means (Internet and SNS) [8, 9]. Another element of this definition is that it can be repetitive and ongoing [8, 9], and may incorporate the component of defenselessness on the part of the victim [7]. Cyberbullying covers various acts, such as sending aggressive and threatening messages, social exclusion, spreading rumors and online identity theft [8–10]. A prominent example is the use of SNS to publish private, inappropriate or humiliating information [1, 10].

The prevalence of cyberbullying varies according to the operational definition employed and the age group under study. Reviews suggest that cyber victimization ranges from 6.5 to 72% worldwide [7–9, 11]. In Europe, rates of cyberbullying vary substantially between studies [12–14]. Ortega and colleagues [12] and Genta and colleagues [13] using the same sample from the DAPHNE II program compared emotional reactions and cyberbullying involvement rates between Spain, the United Kingdom and Italy, obtaining rates of 6.2, 4 and 5.4%, respectively. The “EU Kids Online” study of over 25,000 children between the ages of 9 and 16 reported prevalence of cybervictimization ranging between 2 and 14%, lowest in Italy and Portugal and highest in Estonia and Romania [14, 15]. The “Net Children Go Mobile” project found higher rates, increasing from 7 to 12% from 2010 to 2014 [16]. National studies in individual European countries have found rates of cyberbullying victimization from 5.5 to 44% [12, 17–25].

### Association with psychosocial characteristics

Victims of cyberbullying report an array of psychopathological symptoms, including internalizing problems [26, 27] such as feelings of loneliness, depression [28] and lack of self-esteem [10, 29]. Tsitsika and colleagues [27] indicated that cyberbullying has a consistent effect on psychosocial wellbeing in all countries studied, although varying in magnitude. Moreover, it has been related to social anxiety and isolation [11, 30], as well as to externalizing problems [27], illicit drug use and conduct problems [7, 8, 31].

### Risk factors for cyberbullying

#### Socio-demographic risk factors and cultural differences

Gender and age are the most prominent of the few socio-demographic variables that have been studied as potential risk factors associated with cyberbullying victimization, with rather inconsistent results [9, 32]. Whereas the literature on traditional bullying regards females as greater victims of relational aggression, no such association has been clearly established in cyberbullying research [3, 7, 8, 13, 24, 29, 33]. Some studies have detected an inverse relationship between age and cyberbullying victimization [24, 34], possibly due to the greater impulsivity of younger individuals and their increased likelihood of using SNS to meet new people [10], as well as a peak of cybervictimization amongst younger adolescents [24, 34]. Higher rates of cyberbullying have been reported among those whose parents’ educational level was low or middle compared to high [27]. Finally, family relationships and family structure (e.g. single-parent family), as the key factors in an adolescent’s “micro” developmental context, may be related to experiencing cybervictimization as they influence family cohesion (i.e. “emotional bonding” between family members), and the provision of support and engagement in shared activities between parents and adolescents [35]. Absence of family communication seems to be associated with an inability of cybervictims to open up about their experience to their parents [35–39].

#### Internet use and SNS variables

Communication with parents, including parental monitoring of Internet use, has been repeatedly studied in association with cyberbullying [4, 14, 35, 39–41]. Restrictive mediation (control of time spent online, content filtering and monitoring) seems to be resented by adolescents [35, 42, 43]. However, active mediation is considered protective not only in increasing safety but also in buffering negative psychological impacts on the victim, by increasing the likelihood of their confiding in their parents in the event of victimization [4, 36]. “EU Kids Online” appears to be the only cross-national study that has investigated parental monitoring in relation to cyberbullying, finding it to be a protective factor for online risks [14, 44]. Regarding the extent of adolescent Internet and SNS use, higher levels have been associated with increased risk of cybervictimization [4].

### Theoretical framework and hypothesis

A child’s social environment plays a fundamental role in determining the level of risks online, including cybervictimization [14, 16, 45]. A bio-ecological view of human development posits that a child’s online experience is shaped by multiple layers of interacting environmental systems [16, 46, 47]. Using a child-centered perspective [14] in the study of these systems requires the exploration of “within-person competencies”, “interpersonal variables” (family and peers) and “extra-personal” context-dependent variables (social and cultural) in order to account for risk and protective factors that may correlate with cybervictimization, [16, 45, 48]. A contextual and comparative approach may allow an understanding of how national and cultural characteristics affect a child’s Internet use on a continuum of different levels of directedness.

The abovementioned prevailing social and cultural structures seem to have a profound effect on the phenomenon of cyberbullying in adolescence, but have not yet been analyzed in terms of country-specific differences [3, 13]. Past literature has extensively discussed psychological profiles of bullies and victims, and compared cross-national prevalence rates, but little is known about the factors associated with the likelihood of cyberbullying victimization in different European countries. It is clear that countries have experienced diverse rhythms in the development and integration of information communication technologies (ICT), and along with social and cultural differences across nations it is likely that the factors related to cybervictimization are not consistent throughout Europe. For this purpose, the present study within the context of the EU.NET.ADB project [48] aimed to compare patterns of cyberbullying victimization across seven European countries. An exploratory goal unique to this study was to identify differences in demographic (gender, age, parental educational level, age of first contact with Internet) and family factors (family structure, parental mediation) that increase the odds of the occurrence of online victimization in the countries studied. It was hypothesized that daily use of SNS and the Internet would increase odds of cybervictimization cross-nationally, in line with the hypothesis that increased usage is associated with increased online risks [14, 35]. Following previous research noting the emotional impact of cyberbullying [14, 27], it was expected that internalizing and externalizing difficulties would increase the odds of cybervictimization across countries.

## Methods

### Procedure and participants

This cross-sectional, quantitative, school-based study was performed in the context of the EU.NET.ADB protocol [48], in seven European countries (Germany, Greece, Iceland, the Netherlands, Poland, Romania, Spain), during the school year 2011–2012. The study and its methods received ethical approval according to the requirements of each participating country [48]. A common research protocol was employed by all countries. A random clustered probability sample of adolescents attending school in the 9th and 10th grades was drawn in each country. The primary sampling unit (PSU) was the school class and official national lists were used as sampling frames, stratified according to region (using the European Union NUTS system or other appropriate national regional classification) and population density. A random sample of about 100 classes was drawn in each country in order to achieve a target sample size of 2000 adolescents. These classes were selected by systematic sampling from the list commencing from a random starting point. In case of non-class-based educational systems, clusters were formed in line with current school structure, and a similar sampling procedure was followed [48]. In the absence of a list, classes were selected with probability proportional to size. These procedures resulted in the selection of individual adolescents with equal probabilities.

All students registered in the selected classes were eligible for participation. All participants were required to provide parental informed consent; forms that emphasized the confidentiality and anonymity of the study were provided to their legal guardians prior to the execution of the study. Students attending class on the day of data collection completed anonymously a paper-and-pencil self-report questionnaire administered by a trained researcher in one school hour. The anonymous and confidential nature of the study was stressed. Further details of methodology can be found in the EU.NET.ADB project report [48].

The questionnaire was completed by 13,708 adolescents. Approximately 10% of registered students were absent on the day of data collection and 3% of those present either refused to participate or did not have the necessary permission. The response rate, as a percentage of registered students, was very high in all countries (from 95.0% in Spain to 86.7% in Romania) except Iceland (62.9%). Of those who completed the questionnaire, 129 who fell outside the 14–17 years age range and 295 who did not state their age or gender were eliminated. Of the remaining 13,284, 912 (6.9%) did not answer the question about cyberbullying. This left a total sample of 12,372 14–17-year-olds available for the present analysis.

### Materials

#### Socio-demographic characteristics

The socio-demographic variables that were investigated for associations with the experience of cyberbullying were age, gender, parental educational level (low/middle vs. high) and parental marital status (married/living together vs. separated/divorced/single-parent family). Parental educational level, which served as proxy for social class, was defined as the higher of the levels achieved by the two parents. Among Internet-related behaviors, participants were asked about their age at starting use of new technologies, daily amount of SNS use, parents’ permission for content visited on the Internet (adolescent’s agreement with the statement “My parents allow me to visit every website that I want”) and parental control of time spent online (response to the question “How often do your parents say that you are only allowed to go on the Internet until a certain time?”). Adolescents were asked to report their SNS and Internet use on a typical day in the past 12 months, with responses ranging from “not at all” to “more than 4 hours.” The weighted average of use during weekdays and weekends provided a single weekly estimate, which was dichotomized at the median into moderate SNS use (< 2 h daily) and heavier SNS use (> 2 h daily) [27].

#### Cyberbullying victimization

The following detailed, yet simply phrased, introductory definition and examples of bullying behaviors online were provided in the questionnaire, with emphasis on the elements of hostility or hurtfulness, the repetitiveness of the event and the use of the Internet as the medium of choice [27]: “Sometimes children or teenagers can do hurtful or nasty things to someone and this can often be quite a few times on different days over a period of time, for example. This can include: teasing someone in a way this person does not like; spreading false/malicious rumors; sending someone mean or threatening messages; systematically excluding, ignoring, and isolating. When people are hurtful or nasty to someone in this way, it can happen on the Internet (e-mail, instant messaging, social networking, chat rooms)”. This comprehensive description was chosen instead of using the term “cyberbullying” due to the potential perceived ambiguity regarding the behaviors it encompasses and the absence of equivalent translations in some countries [27, 49]. Subsequently participants were asked the following question: “Has someone acted in this kind of hurtful or nasty way to you in the past 12 months on the Internet?” with possible responses being “no”, “yes” and “do not know/prefer not to say”. This question was adapted from the “EU Kids Online” study [14] due to its cultural adaptation and translation of high quality [48], and was partly in line with Olweus’ Bully/Victim questionnaire [27, 48].

#### Psychosocial wellbeing

Psychosocial wellbeing was measured using the Youth Self-Report (YSR) [50]. The YSR is an instrument measuring adolescent competence and problems in social, academic, cognitive, internalizing and externalizing behaviors [50]. It had already been translated and standardized in all participating countries, and is known to present excellent psychometric properties and suitability for use in cross-national research [51, 52]. This instrument was not employed in the German arm of the study.

### Data analysis

Variables pertaining to the socio-demographic and Internet use related variables were expressed as absolute and relative frequencies. Multiple logistic regression analysis was used in order to find factors independently associated with the likelihood of cyberbullying separately in each country. Adjusted odds ratios with 95% confidence intervals were computed from the results of the logistic regression analyses. All *p*-values reported are two-tailed. All statistical tests, standard errors and confidence intervals were corrected for the complex sample design with countries as strata and classes as clusters, using the Complex Samples procedure of SPSS statistical software (version 19.0).

## Results

Sample characteristics are shown in Table 1. From the total sample, 46.8% of the participants were male and 38.4% aged from 16 to 17.9 years. Parental educational level was high in 62.8% of the participants and in most cases (80.1%) parents were married or lived together. Daily use of SNS for more than two hours was reported by 36.4% of the adolescents and daily Internet use for more than two hours was reported by 51.0%. In most cases (77.9%) the adolescents declared that their parents often or very often allowed them to visit every site and 54.1% reported that their parents never or seldom told them to stay on the Internet for a certain time.

**Table 1:** Sample characteristics

	Ν (%)
Total sample	12,372 (100)
Country	
Germany	2178 (17.6)
Greece	1894 (15.3)
Iceland	1790 (14.5)
Netherlands	1176 (9.5)
Poland	1849 (14.9)
Romania	1612 (13.0)
Spain	1873 (15.1)
Gender	
Females	6586 (53.2)
Males	5786 (46.8)
Age (years)	
14–15.9	7627 (61.6)
16–17.9	4745 (38.4)
Parental educational level	
Low/middle	3903 (37.2)
High	6603 (62.8)
Parental family status	
Married/ living together	9662 (80.1)
Divorced/ separated/ single-parent family	2399 (19.9)
Age at first starting to use the Internet: mean years (SD)	9.6 (2.5)
Daily use of SNS	
No use/< 2 h	7411 (63.6)
≥ 2 h	4250 (36.4)
Daily Internet use	
No use/< 2 h	5635 (49.0)
≥ 2 h	5874 (51.0)
Parents allow me to visit every site	
Never/ seldom	1186 (10.3)
Sometimes	1351 (11.8)
Often/ very often/ my parents do not know which websites I visit	8928 (77.9)
How often parents say to stay on Internet a certain time	
Never/ seldom	6652 (54.1)
Sometimes	3031 (24.6)
Often/ very often	2621 (21.3)
Internalizing Problems score, mean (SD)	10.9 (8.6)
Externalizing Problems score, mean (SD)	11.5 (8.5)

**Fig. 1:**
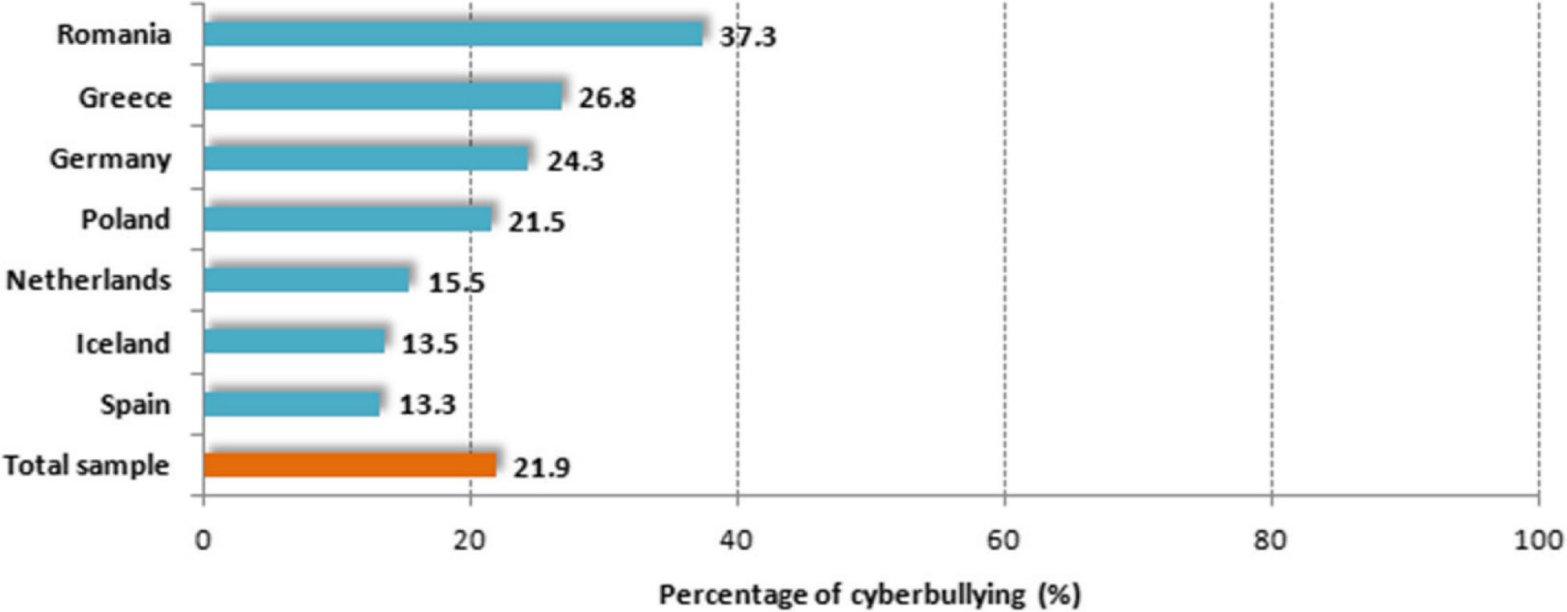
Proportion of adolescents that has been bullied online in past 12 months, by country

Figure 1 presents the proportion of youth that has been bullied online in past 12 months, by country. The highest rates occurred in Romania (37.3%), Greece (26.8%) and Germany (24.3%), following by Poland (21.5%). The lowest rates were found in the Netherlands, Iceland and Spain (see Fig. 1). Table 2 shows the rate of cyberbullying within each category of the sociodemographic and Internet-related variables studied, separately in each country. The crude odds ratios computed from these data are shown in Tables 3 and 4, which also present the adjusted odds ratios from the multiple logistic regression analyses with cyberbullying victimization as the dependent variable. Among the socio-demographic and Internet-related variables studied, parental education level and age at first Internet use were not independently associated (at *p* = 0.05) with cyberbullying victimization in any of the countries. Increased Internalizing and Externalizing Problems were associated with greater odds of cybervictimization everywhere (Table 3), except for Externalizing problems in Romania. Additionally in Romania, adolescents over 16 years old had 48% lower odds of being a victim of cyberbullying compared to younger adolescents, and daily use of SNS and the Internet for more than two hours was associated in Romania with 83 and 57% greater probability for victimization, respectively. In Poland, daily use of SNS for more than two hours was associated with 53% greater odds of cyberbullying victimization in the past twelve months, and the odds of being bullied online also increased when parents aimed at controlling the time of Internet use sometimes (49% greater odds) or very frequently (63%) (Table 3).

**Table 2:** Prevalence (with 95% confidence interval) of ever having been the victim of cyberbullying in relation to sociodemographic and internet use factors, separately in each country

	Greece	Spain	Romania	Poland	Germany	Netherlands	Iceland
Gender							
Females	28.9% (284/982)^a^ 26.1–31.9	15.7% (153/977) 12.9–18.9	39.0% (358/917) 35.6–42.6	23.4% (228/973) 20.6–26.7	24.7% (295/1196) 21.9–27.7	19.4% (116/597) 16.2–23.1	16.0% (151/944) 13.7–18.6
Males	24.6% (224/912) 21.8–27.6	10.7% (96/896) 8.5–13.4	35.1% (244/695) 31.0–39.5	19.4% (170/876) 16.5–22.6	23.9% (235/982) 21.0–27.1	11.4% (66/579) 9.1–14.1	10.8% (91/846) 8.8–13.1
Age (years)							
14–15.9	26.4% (351/1330) 24.0–29..0	12.6% (154/1226) 10.5–14.9	42.6% (179/420) 37.2–48.2	21.1% (288/1368) 18.4–23.9	24.3% (304/1249) 21.6–27.2	16.3% (74/455) 13.3–19.7	13.4% (211/1579) 11.5–15.5
16–17.9	27.8% (157/564) 24.4–31.5	14.7% (95/647) 11.8–18.1	35.5% (423/1192) 32.4–38.7	22.9% (110/481) 19.0–27.3	24.3% (226/929) 21.7–27.2	15.0% (108/721) 12.3–18.1	14.7% (31/211) 10.7–19.9
Parental educational level							
Low/Middle	29.1% (229/787) 26.1–32.3	13.8% (93/672) 10.9–17.4	38.7% (273/705) 34.2–43.5	20.4% (147/721) 17.2–24.0	25.0% (133/532) 21.5–28.8	15.6% (27/173) 11.2–21.4	14.1% (44/313) 9.9–19.5
High	25.0% (249/997) 22.4–27.7	13.1% (132/1005) 10.9–15.8	38.7% (276/714) 35.4–42.0	23.5% (170/722) 20.1–27.4	24.4% (303/1242) 21.9–27.1	14.7% (106/722) 11.9–18.0	13.6% (163/1201) 11.6–15.8
Parental family status							
Married/ living together	25.5% (409/1602) 23.5–27.6	12.4% (195/1570) 10.5–14.6	36.5% (491/1345) 33.8–39.3	21.0% (309/1470) 18.4–23.9	22.8% (350/1537) 20.4–25.3	14.2% (121/853) 11.8–17.0	12.1% (156/1285) 10.2–14.5
Divorced/ separated/ Single- parent family	35.7% (94/263) 30.7–41.1	17.4% (50/288) 13.2–22.5	41.7% (101/242) 35.6–48.2	23.2% (76/328) 18.9–28.1	28.0% (148/528) 24.5–31.9	19.2% (55/287) 14.3–25.2	17.5% (81/463) 13.9–21.8
Daily use of SNS							
No use/< 2 h	20.9% (252/1203) 18.7–23.4	10.5% (120/1140) 8.5–12.9	30.0% (273/910) 26.1–34.2	18.9% (239/1264) 16.2–21.9	20.0% (235/1173) 17.7–22.6	13.0% (86/664) 10.7–15.6	9.6% (102/1057) 7.7–12.0
≥ 2 h	38.1% (243/637) 34.7–41.7	17.5% (116/661) 14.6–21.0	48.1% (289/601) 44.5–51.7	28.0% (143/510) 24.3–32.1	29.8% (249/835) 26.7–33.1	19.5% (78/400) 15.9–23.7	20.3% (123/606) 17.1–23.9
Daily Internet use							
No use/< 2 h	21.4% (234/1095) 18.9–24.1	10.6% (118/1112) 8.5–13.2	29.9% (217/726) 25.8–34.3	19.1% (164/858) 16.2–22.5	20.6% (209/1015) 17.8–23.7	12.0% (55/458) 9.4–15.3	8.3% (59/707) 6.3–10.9
≥ 2 h	34.5% (261/756) 31.2–38.0	17.5% (126/720) 14.9–20.4	44.0% (356/810) 40.7–47.3	23.7% (218/921) 20.7–27.0	27.4% (287/1046) 24.7–30.4	18.1% (118/652) 15.0–21.7	16.6% (164/989) 13.9–19.6
Parents allow me to visit every site							
Never/ seldom	25.7% (69/268) 21.2–30.8	14.4% (28/195) 9.7–20.8	24.2% (39/161) 17.1–33.1	16.3% (25/153) 11.0–23.6	30.7% (39/127) 23.2–39.4	13.7% (10/73) 7.4–24.1	12.4% (26/209) 8.7–17.4
Sometimes	25.2% (85/337) 20.0–31.0	10.0% (22/221) 6.5–15.0	32.2% (75/233) 26.1–38.9	14.8% (20/135) 9.6–22.1	21.0% (34/162) 15.7–27.5	16.2% (11/68) 9.1–27.1	14.4% (28/195) 10.2–19.8
Often/ very often/ parents don’t know	27.7% (352/1271) 25.3–30.2	13.7% (199/1449) 11.8–15.9	34.9% (155/444) 30.8–39.3	22.6% (351/1553) 20.1–25.4	24.2% (444/1835) 22.1–26.4	15.4% (156/1010) 13.1–18.1	13.4% (183/1366) 11.4–15.7
Parents restrict time on Internet							
Never/ seldom	25.3% (180/712) 22.0–28.8	13.0% (78/601) 10.3–16.2	35.4% (344/971) 32.3–38.7	19.5% (208/1069) 16.6–22.6	23.3% (332/1424) 20.8–26.0	14.5% (122/843) 12.1–17.3	13.0% (134/1032) 10.4–16.0
Sometimes	27.9% (138/495) 23.9–32.3	12.7% (67/526) 9.6–16.8	38.5% (160/416) 33.5–43.6	23.1% (110/476) 19.2–27.5	23.8% (103/432) 19.7–28.6	15.2% (32/211) 11.0–20.5	13.7% (65/475) 10.4–17.8
Often/ very often	28.0% (188/672) 24.7–31.5	14.1% (104/740) 11.5–17.0	45.0% (98/218) 38.6–51.5	26.4% (78/295) 21.2–32.5	29.7% (92/310) 25.0–34.8	24.1% (27/112) 16.5–33.9	14.6% (40/274) 11.3–18.7

^a^In parentheses, number bullied / sample size

**Table 3:** Cyberbullying in relation to sociodemographic and other factors in Greece, Spain, Romania and Poland separately: crude odds ratio (OR) and adjusted OR from multiple logistic regression analyses, with 95% confidence intervals

		Greece			Spain			Romania			Poland	
	OR 95% CI	Adjusted OR	P	OR 95% CI	Adjusted OR	P	OR 95% CI	Adjusted OR	P	OR 95% CI	Adjusted OR	P
Gender												
Females	1^b^	1^b^		1^b^	1^b^		1^b^	1^b^		1^b^	1^b^	
Males	0.80 (0.64–0.99)	0.79^a^ (0.62-1.03)	0.08	0.65 (0.46–0.90)	0.79 (0.57–1.11)	0.18	0.85 (0.67–1.06)	1.06 (0.73–1.53)	0.77	0.79 (0.63–0.98)	1.09 (0.79–1.49)	0.61
Age (years)												
14–15.9	1	1		1	1		1	1		1	1	
16–17.9	1.08 (0.86–1.35)	1.08 (0.84–1.4)	0.55	1.20 (0.91–1.58)	1.26 (0.91–1.74)	0.16	0.74 (0.57–0.96)	0.52 (0.35–0.75)	0.001	1.11 (0.87–1.43)	1.05 (0.75–1.46)	0.78
Parental educational level												
Low/Middle	1	1		1	1		1	1		1	1	
High	0.81 (0.67–0.99)	0.94 (0.74–1.2)	0.64	0.94 (0.68–1.31)	1.15 (0.83–1.6)	0.39	1.00 (0.79–1.26)	1.14 (0.80–1.62)	0.47	1.20 (0.93–1.55)	1.10 (0.82–1.49)	0.52
Parental family status												
Married/ living together	1	1		1	1		1	1		1	1	
Other	1.62 (1.27–2.07)	1.36 (0.98–1.87)	0.063	1.48 (1.06–2.08)	1.33 (0.9–1.97)	0.16	1.25 (0.97–1.59)	1.06 (0.67–1.70)	0.80	1.13 (0.86–1.49)	1.00 (0.68–1.47)	0.99
Age at first Internet use	0.97 (0.92–1.01)	0.99 (0.93–1.05)	0.77	0.99 (0.92–1.06)	0.99 (0.92–1.07)	0.79	0.96 (0.92–1.00)	1.07 (0.99–1.17)	0.08	0.97 (0.91–1.02)	1.04 (0.96–1.12)	0.39
Daily use of SNS												
No use/< 2 h	1	1		1	1		1	1		1	1	
≥ 2 h	2.33 (1.87–2.89)	1.34 (0.94–1.91)	0.10	1.81 (1.35–2.43)	0.96 (0.60–1.52)	0.86	2.16 (1.71–2.73)	1.83 (1.21–2.76)	0.004	1.67 (1.32–2.12)	1.53 (1.05–2.24)	0.028
Daily Internet use												
No use/< 2 h	1	1		1	1		1	1		1	1	
≥ 2 h	1.94 (1.55–2.43)	1.34 (0.95–1.90)	0.10	1.79 (1.36–2.34)	1.34 (0.85–2.10)	0.21	1.84 (1.45–2.33)	1.57 (1.02–2.42)	0.041	1.31 (1.06–1.62)	0.98 (0.69–1.41)	0.93
Parents allow me to visit every site												
Never/ seldom	1	1		1	1		1	1		1	1	
Sometimes	0.97 (0.67–1.41)	1.16 (0.75–1.77)	0.51	0.66 (0.38–1.16)	0.89 (0.45–1.76)	0.75	1.49 (0.88–2.51)	1.28 (0.73–2.22)	0.39	0.89 (0.50–1.58)	0.85 (0.37–1.96)	0.71
Often/ very often/ parents don’t know	1.11 (0.84–1.45)	1.08 (0.74–1.56)	0.70	0.95 (0.61–1.50)	1.05 (0.62–1.76)	0.87	1.68 (1.07–2.63)	1.29 (0.75–2.21)	0.36	1.50 (0.94–2.38)	1.27 (0.69–2.33)	0.44
Parents limit time on Internet												
Never/ seldom	1	1		1	1		1	1		1	1	
Sometimes	1.14 (0.97–1.51)	1.17 (0.87–1.58)	0.31	0.98 (0.67–1.43)	1.13 (0.75–1.70)	0.56	1.14 (0.90–1.45)	1.11 (0.74–1.64)	0.62	1.24 (0.94–1.66)	1.49 (1.06–2.10)	0.022
Often/ very often	1.15 (0.90–1.47)	1.2 (0.91–1.59)	0.19	1.10 (0.80–1.51)	1.18 (0.81–1.73)	0.39	1.49 (1.13–1.96)	1.32 (0.81–2.16)	0.27	1.49 (1.09–2.02)	1.63 (1.08–2.47)	0.021
Internalizing Problems	1.06 (1.05–1.07)	1.04 (1.02–1.05)	< 0.001	1.08 (1.06–1.10)	1.06 (1.04–1.08)	< 0.001	1.07 (1.05–1.09)	1.05 (1.02–1.08)	0.004	1.05 (1.04–1.07)	1.03 (1.01–1.05)	0.005
Externalizing Problems	1.06 (1.04–1.07)	1.04 (1.02–1.05)	< 0.001	1.06 (1.04–1.09)	1.04 (1.02–1.06)	< 0.001	1.06 (1.04–1.07)	1.02 (1.00–1.05)	0.06	1.07 (1.05–1.08)	1.05 (1.02–1.07)	< 0.001

^a^Odds Ratio (95% Confidence Interval) adjusted for all other variables listed

^b^indicates reference category

**Table 4:** Cyberbullying in relation to sociodemographic and other factors in Germany, the Netherlands and Iceland separately: crude odds ratio (OR) and adjusted OR from multiple logistic regression analyses, with 95% confidence intervals

	Germany			Netherlands			Iceland		
	OR 95% CI	Adjusted OR	P	OR 95% CI	Adjusted OR	P	OR 95% CI	Adjusted OR	P
Gender									
Females	1^b^	1^b^		1^b^	1^b^		1^b^	1^b^	
Males	0.96 (0.76–1.21)	1.03^a^ (0.80–1.33)	0.82	0.53 (0.39–0.73)	0.50 (0.30–0.83)	0.008	0.63 (0.51–0.79)	0.75 (0.5–1.12)	0.16
Age (years)									
14–15.9	1	1		1	1		1	1	
16–17.9	1.00 (0.82–1.22)	1.04 (0.81–1.34)	0.74	0.91 (0.68–1.21)	0.85 (0.54–1.33)	0.47	1.12 (0.79–1.59)	0.99 (0.58–1.66)	0.96
Parental educational level									
Low/middle	1	1		1	1		1	1	
High	0.97 (0.78–1.21)	0.95 (0.72–1.25)	0.70	0.93 (0.58–1.48)	1.21 (0.69–2.13)	0.51	0.96 (0.63–1.46)	1.59 (1.00–2.51)	0.048
Parental family status									
Married/ living together	1	1		1	1		1	1	
Other	1.32 (1.06–1.65)	1.26 (0.96–1.66)	0.10	1.43 (0.95–2.18)	1 (0.61–1.64)	0.99	1.54 (1.09–2.16)	1.25 (0.86–1.83)	0.25
Age at first Internet use	1.00 (0.96–1.05)	0.97 (0.91–1.03)	0.32	0.97 (0.89–1.05)	0.93 (0.83–1.05)	0.25	0.97 (0.91–1.04)	0.98 (0.90–1.07)	0.61
Daily use of SNS									
None/< 2 h	1	1		1	1		1	1	
≥ 2 h	1.70 (1.38–2.08)	1.66 (1.18–2.34)	0.004	1.63 (1.19–2.24)	1.15 (0.68–1.95)	0.61	2.38 (1.77–3.21)	1.37 (0.89–2.13)	0.16
Daily Internet use									
None/< 2 h	1	1		1	1		1	1	
≥ 2 h	1.46 (1.16–1.83)	0.91 (0.64–1.29)	0.60	1.62 (1.14–2.30)	1.23 (0.71–2.12)	0.46	2.18 (1.56–3.06)	1.46 (0.91–2.34)	0.11
Parents allow me to visit every site									
Never/ seldom	1	1		1	1		1	1	
Sometimes	0.60 (0.38–0.95)	0.52 (0.26–1.04)	0.06	1.22 (0.46–3.25)	0.79 (0.20–3.10)	0.73	1.18 (0.68–2.04)	1.25 (0.61–2.56)	0.55
Often/ yery often/parents don’t know	0.72 (0.49–1.06)	0.61 (0.36–1.04)	0.07	1.15 (0.59–2.24)	1.05 (0.38–2.84)	0.93	1.09 (0.74–1.61)	0.76 (0.42–1.37)	0.36
Parents limit time on Internet									
Never/ seldom	1	1		1	1		1	1	
Sometimes	1.03 (0.78–1.37)	1.21 (0.88–1.66)	0.24	1.06 (0.70–1.59)	0.80 (0.42–1.5)	0.48	1.06 (0.72–1.57)	1.28 (0.86–1.92)	0.23
Often/ Very often	1.39 (1.06–1.82)	1.37 (0.96–1.96)	0.08	1.88 (1.14–3.09)	1.66 (0.86–3.21)	0.13	1.15 (0.77–1.70)	1.13 (0.69–1.85)	0.64
Internalizing Problems	-^c^	-^c^		1.07 (1.05–1.10)	1.04 (1.01–1.07)	0.004	1.08 (1.07–1.10)	1.05 (1.02–1.07)	< 0.001
Externalizing Problems	-^c^	-^c^		1.05 (1.03–1.07)	1.03 (1–1.06)	0.028	1.10 (1.08–1.12)	1.07 (1.05–1.10)	< 0.001

^a^Odds Ratio (95% Confidence Interval) adjusted for all other variables listed

^b^indicates reference category

^c^The YSR was not administered in Germany

Multiple logistic regression analysis for cyberbullying victimization in Germany (Table 4) indicated that daily use of SNS for more than two hours was independently associated with cyberbullying victimization, increasing the odds of its occurrence by 66%. In the Netherlands, males had only half the odds of having been a victim of cyberbullying compared to females, while in Iceland the odds of cyberbullying were 59% higher in adolescents whose parents had high educational level (Table 4).

## Discussion

The present study investigated cyber victimization in adolescents from seven European countries. Previous research has focused primarily on cyberbullying victimization prevalence rates and related psychopathological symptoms [6, 11, 12, 30, 31, 53]. Consequently, the present study is novel in comparing individual factors associated with cyberbullying victimization, namely socio-demographic, Internet use and psychosocial variables, between countries. On the whole, the pattern of victimization rates across countries is consistent with the “EU Kids Online” study [14], with the exception of Greece which held a higher ranking by percentage of adolescents victimized. However, the current study found overall higher rates of cybervictimization compared to EU Kids Online, ranging from 13.3 to 37.3%.

The relatively high percentages recorded in the present study may reflect a growing temporal trend, but could also be influenced by differences in the ages of participants between studies. The “EU Kids Online” sample consisted of 9–16-year-old adolescents, whereas the EU.NET.ADB study included adolescents aged from 14 to 17 - a population much more likely to use the Internet and SNS actively and more frequently, and in which ICT knowledge is often accompanied by a tendency for online bullying of peers [6]. Furthermore, data collection procedures differed between the two studies.

### Results and differences by country

#### Romania

Romania presented the highest rates of cyberbullying, supporting previous literature that has consistently ranked it highest in its rate of cybervictimization along with other Eastern European countries [6, 14]. The percentage of Romanian adolescents bullied online was much greater than that reported in the “EU Kids Online” study [14]. Romania has seen a sharp increase in Internet and SNS use in recent years; underestimation of risks by parents, and absence of parental digital skills may have contributed to the emergence of high rates of cyberbullying victimization [44]. This sudden rise in Internet use, compared to the steadier and much earlier growth of Internet use in the other European countries studied [44], leaves a large technological gap between parents and the younger generation. Moreover, the absence of a legislative framework for the protection of Romanian children online, as well as the current lack of integration of ICT components into education, limits the promotion of online safety and awareness of online risks [54].

#### Geece

Greece was the only country that deviated from the pattern established by the “EU Kids Online” study [14] and other national studies [20, 21, 25], presenting higher rates of cybervictimization compared to previous studies [9]. While Greece has made several efforts regarding promoting online safety, the very rapid diffusion of Internet technologies has reached many adolescents who lack digital skills and awareness of online risks. In particular, 38% of adolescents leave their Facebook profile public [44]. However, this growing pattern, along with internalizing and externalizing problems arising as the only associated factors, may reflect the deeply rooted contemporary problems Greece is facing, which have affected adolescents on a national and societal level [55]. The socioeconomic crisis has influenced adolescent development in emotional and social domains, influencing both their own behavior but also the interacting levels of their socio-emotional development; it has affected the way they cope with their everyday concerns and their family’s financial limitations [56], making them more prone to violence [55], and influencing their sensitivity to stressors, one of which could be online victimization.

#### Poland

The percentage of adolescents that had experienced cyberbullying in Poland was similar to rates reported elsewhere in Europe [9]. In contrast to Romania and Greece, Poland has enforced legislation targeting cyberbullying as a form of emotional violence and has included ICT literacy in the primary and secondary school curricula [57]. All the same, rates of cyberbullying victimization in this study were greater than those reported in the “EU Kids Online” study [14]. Furthermore, in Poland, attempts at parental control of time spent online were established as an individual factor significantly associated with cyberbullying victimization, supporting previous research that has repeatedly associated it with cyberbullying [4, 40, 41]. These results are in contrast with the “EU Kids Online” study, which reported that 74% of children have a positive attitude towards parental mediation - which tends to be of a restrictive nature – based, however, on an overall younger sample [44].

#### Gemany

In the present study rates of cyber victimization in Germany were higher than in past national studies and European research programs, with the exception of a study conducted by Katzer and colleagues [22] on chat room victimization. Specifically, Riebel and colleagues [23] found an overall cyberbullying victimization rate of 14.1%. The “EU Kids Online” study estimated cyberbullying among German children and adolescents at 5% [14]. In contrast to Romania and Greece, Germany has made substantial efforts to promote online safety, both through the “Internet and Digital Society Committee of Inquiry” and individual states, including the “Medienpass (media passport) NRW” program, an attempt to improve education on digital skills by offering advice and guidance [58].

#### Te Netherlands

The Netherlands emerged as one of the countries with the lowest rates of cybervictimization, although the rate was higher than the 4% reported for Dutch children in the “EU Kids Online” study [14]. According to a qualitative study conducted in the Netherlands by Jacobs and colleagues [59], cyber-bullies are judged as “cowards” by their peers and are heavily criticized amongst young students, which may account for the low rates reported. Furthermore, the Netherlands has previously been reported as a country with considerable levels of ICT literacy that were established as early as 2004, allowing for the integration of digital skills into education and the use of active parental monitoring that may contribute to the lower rates observed [44].

#### Iceland

In Iceland, the low rates of cyberbullying victimization are difficult to compare, because findings establishing national rates are scarce. Higher educational level was associated with increased odds of cybervictimization. This may be explained by the greater use and potentially increased access to electronic equipment by adolescents who are in better socioeconomic circumstances [14]. However, it also suggests that educational attainment is not necessarily related to parental digital skills which may contribute to adolescent’s safer use of Internet technologies.

#### Spain

The present study found a higher probability of adolescents in Spain being cyber-bullied than in a number of previous Spanish national studies, which obtained estimates of 5.5 to 9.3% [53], and in European cross-national studies [13, 14]. However, the rate was lower than in one study conducted by Buelga and colleagues [60], who reported rates of 29% for Internet victimization in the past 12 months. The identification of internalizing and externalizing problems as main risk factors demonstrates that socio-emotional and behavioral traits of victims [31, 53] are associated with the experience of cyberbullying. Regarding gender, our findings conflict with some Spanish studies that highlighted the greater rates of victimization among females [13, 53], but support research that found low predictive power of gender and age in predicting cyberbullying [32].

### Cybervictimization in relation to socio-demographic characteristics

Although previous studies compared gender and age in relation to the occurrence of different types of cyberbullying [6, 13], the present study’s novelty is rooted in its comparative approach to establishing socio-demographic risk factors cross-nationally in this age group. Regarding gender differences, results from the majority of countries are in agreement with previous literature that did not find gender to be statistically significantly associated with cyberbullying [7–9, 14], demonstrating that in terms of victimization (not necessarily perpetration) girls and boys can equally be recipients of relational aggression online. Consequently, it is imperative to develop prevention measures that acknowledge members of either gender as potential victims of bullying online.

In the Netherlands, however, girls were twice as likely to experience cyberbullying compared to boys, in line with past research that established greater cyberbullying victimization prevalence in girls than boys [3, 14]. This result may be to some extent attributed to the definition employed and the perception of Dutch adolescents, and suggests a limitation of the operational definition employed; specifically, it has been reported that girls in the Netherlands experience online victimization as more negative than boys and older students [44]. Consequently, the use of the terms “hurtful” and “nasty” in the operationalization of cyberbullying may account for the higher odds of female victimization in the Netherlands.

Finally, age arose as a risk factor of cyber victimization only in Romania [27], reflecting the inconsistent results obtained in the literature [8, 22, 24, 34]. Since this finding was only established in Romania, it is worth considering these results in light of SNS and Internet usage being associated with cyberbullying victimization, possibly suggesting that absence of computer literacy over safety issues in younger adolescents in Romania may play an important role in their risk of victimization. Future research should thus be directed at observing patterns and means of cyberbullying victimization in relation to age.

### Association with psychosocial characteristics

Going beyond previous studies merely reporting the prevalence of cybervictimization, we carried out regression analyses relating cybervictimization to the adolescent’s psychosocial characteristics. Internalizing problems increased the odds of having experienced cyberbullying in all countries analysed. These results correspond to previous findings that associated cybervictimization with lower self-esteem [22], suicidal ideation [26], social anxiety and depression [53]. This consistent association demonstrates that adolescents who internalize their problems are at greater risk of experiencing cyberbullying [6, 9], a relationship that is likely to be bidirectional in nature. On the one hand, adolescents who experience internalizing difficulties may have a greater likelihood of perceiving a behavior as hurtful, but may also manifest such negative feelings in response to the aggressive behavior. In addition, in the present study, externalizing problems increased the odds of online victimization in Greece, Spain, Poland, the Netherlands and Iceland, consistent with previous literature that establishes a connection between externalizing behavior and cyberbullying victimization [7, 8, 13, 27].

### Internet use and SNS variables

Although Internet use for more than 2 h a day arose as a risk factor for cyberbullying only in Romania, the odds for having experienced cyberbullying increased when SNS were used for more than 2 h a day in Romania, Germany and Poland. This is an important finding, challenging past research that postulates that ownership, rather than use of an SNS profile, increases the risk of cybervictimization [5]. Spending a greater amount of time online, and specifically on SNS, is consistently associated with cyberbullying, possibly due to the increased posting of private information and meeting strangers online [5]. Furthermore, if social media are used as a means of communication within the context of relationships, they also become a vehicle for problem solving and the expression of relational aggression [4]. Either way, increased SNS use places adolescents at greater risk of cyberbullying [14]. Most importantly, the statistical significance of SNS in increasing cyberbullying victimization probability occurred in countries with high rates of cyberbullying, but not in countries with lower rates, despite the high use of SNS in countries such as the Netherlands and Spain [44].

### Strengths and limitations

The primary strength of the present study lies in its novelty in providing estimates of cyberbullying victimization across seven European countries in this specific age group. Furthermore, this study identifies factors that increase the odds of the occurrence of online victimization, including demographic, socio-economic and psychosocial characteristics. The large representative samples and anonymous self-reporting have substantially restricted the potential for biases. However, this study also has certain limitations. Specifically, its cross-sectional nature limits our understanding of the direct causes of cyberbullying victimization, and self-reports may introduce bias if there is an element of social desirability in responding. Furthermore, five years have passed since the study was conducted, thus limiting the extent to which it represents the current picture of cybervictimization, bearing in mind the rapid technological developments in Internet, and especially mobile, technologies. Finally, in common with almost all school-based studies, data were collected from only those students who were present on the day of data collection.

## Conclusion

The present study has mapped out a number of factors that are associated with cyberbullying victimization in seven European countries, pinpointing important social differences that may affect the prevalence of this phenomenon. These findings have important implications for both clinical and educational settings: the psychosocial impact of cyberbullying needs to be acknowledged and dealt with, as it is related to distress and psychopathological symptoms. The absence of studies that could explain different rates of cybervictimization between countries limits our ability to account for the present findings, but also indicates that this field has substantial potential for exploration. Educational settings would benefit from integrating ICT education into their curricula, especially in countries where use of the Internet has risen abruptly. Future research would gain from studying cyberbullying victimization from a socio-ecological perspective, to better establish the relationship of societal norms and macro changes in Internet use behavior to the expression of violence amongst adolescents.
